# Dissecting Seed Mucilage Adherence Mediated by FEI2 and SOS5

**DOI:** 10.3389/fpls.2016.01073

**Published:** 2016-07-29

**Authors:** Jonathan S. Griffiths, Marie-Jeanne Crepeau, Marie-Christine Ralet, Georg J. Seifert, Helen M. North

**Affiliations:** ^1^Institut Jean-Pierre Bourgin, Institut National de la Recherche Agronomique, AgroParisTech, CNRS, Université Paris-Saclay, VersaillesFrance; ^2^Institut National de la Recherche Agronomique, UR 1268 Biopolymères Interactions Assemblages, NantesFrance; ^3^Department of Applied Genetics and Cell Biology, University of Natural Resources and Life Sciences, Vienna, ViennaAustria

**Keywords:** *Arabidopsis*, cell wall, mucilage, cellulose, arabinogalactan protein, receptor-like kinase, polysaccharides, genetics

## Abstract

The plant cell wall is held together by the interactions between four major components: cellulose, pectin, hemicellulose, and proteins. Mucilage is a powerful model system to study the interactions between these components as it is formed of polysaccharides that are deposited in the apoplast of seed coat epidermal cells during seed development. When seeds are hydrated, these polysaccharides expand rapidly out of the apoplastic pocket, and form an adherent halo of mucilage around the seed. In *Arabidopsis*, mutations in multiple genes have similar loss of mucilage adherence phenotypes including *CELLULOSE SYNTHASE 5* (*CESA5*)/*MUCILAGE-MODIFIED 3* (*MUM3*), *MUM5/MUCI21*, *SALT-OVERLY SENSITIVE 5* (*SOS5*), and *FEI2*. Here, we examine the interactions between these factors to better understand how they participate to control mucilage adherence. Double mutant phenotypes indicated that MUM5 and CESA5 function in a common mechanism that adheres pectin to the seed through the biosynthesis of cellulose and xylan, whereas SOS5 and FEI2, encoding a fasciclin-like arabinogalactan protein or a receptor-like kinase, respectively, function through an independent pathway. Cytological analyses of mucilage indicates that heteromannans are associated with cellulose, and not in the pathway involving SOS5 or FEI2. A SOS5 fluorescent protein fusion (SOS5-mCITRINE) was localized throughout the mucilage pocket, consistent with a structural role in pectin adhesion. The relationship between SOS5 and FEI2 mediated mucilage adherence was examined in more detail and while *sos5* and *fei2* mutants show similar phenotypes, key differences in the macromolecular characteristics and amounts of mucilage polymers were observed. FEI2 thus appears to have additional, as well as overlapping functions, with SOS5. Given that FEI2 is required for SOS5 function, we propose that FEI2 serves to localize SOS5 at the plasma membrane where it establishes interactions with mucilage polysaccharides, notably pectins, required for mucilage adherence prior to SOS5 being released into the apoplast.

## Introduction

The plant cell wall is a complex structure composed of interconnected polysaccharides and proteins. Interactions between cell wall components determine cell shape, axis of elongation, and can affect permeability. Most primary cell walls are composed of three types of polysaccharides, cellulose, pectin, and hemicelluloses (Cosgrove, 2014). Primary plant cell walls are constantly reorganized to accommodate growth or to respond to changing environmental conditions. In order to correctly adjust to the external environment plant cells must be able to sense the conditions of the cell wall (Wolf et al., 2012). The specific mechanisms underlying this perception and response network are largely unknown.

Many receptor-like kinase genes (RLK) have been implicated as cell wall sensors. Multiple genes encoding wall-associated kinases (WAKs) are involved in cell wall signaling including *THESEUS1*, *FEI1*, and *FEI2* (Hematy and Höfte, 2008; Xu et al., 2008; Höfte, 2015). FEI1 and FEI2 are transmembrane proteins that have an external leucine-rich repeat (LRR) domain, one transmembrane domain and a cytosolic kinase domain (Xu et al., 2008). In *fei1 fei2* double mutants, there are substantial reductions in root cell expansion when grown in the presence of salt or sucrose (Xu et al., 2008). FEI1and FEI2 have been proposed to be key components of a signaling pathway that monitors cell wall conditions, and can initiate an intracellular response through hormone signaling (Xu et al., 2008; Steinwand et al., 2014). The first step of this signaling pathway is believed to begin with the fasciclin-like arabinogalactan protein (FLA AGP), SALT-OVERLY SENSITIVE 5/FASCICLIN-LIKE ARABINOGALACTAN PROTEIN 4 (SOS5/FLA4; Shi et al., 2003; Xu et al., 2008). FLA AGPs are extracellular proteins that contain FASCICLIN (FAS) domains, are highly glycosylated, and often contain a C-terminal glycophosphatidyl inositol anchor (GPI; Johnson et al., 2003; Seifert and Roberts, 2007). Mutant *sos5* roots have a similar reduction in cell expansion as the *fei1 fei2* double mutant, and a triple *fei1 fei2 sos5* mutant has a non-additive phenotype, suggesting that these genes function together in a pathway regulating cell expansion (Xu et al., 2008). The precise mechanism through which FEIs and SOS5 control cell expansion remains unclear, with suggestions that these proteins might intervene directly in cellulose biosynthesis, or that they control cell wall architecture through pectin (Shi et al., 2003; Xu et al., 2008; Harpaz-Saad et al., 2011; Griffiths et al., 2014; Basu et al., 2015, 2016).

In addition to a role in modulating root cell shape, both FEI2 and SOS5 are required for the adherence of mucilage polysaccharides to the epidermal cells of the seed coat (Harpaz-Saad et al., 2011; Griffiths et al., 2014). Seed coat mucilage is formed from pectin-rich polysaccharides accumulated in the apoplast of the epidermal cell layer (Haughn and Chaudhury, 2005). These polysaccharides expand rapidly upon hydration of mature seeds, and form non-adherent and adherent layers in *Arabidopsis*. The adherent mucilage layer can only be removed with harsh acid treatment, or enzymatic digestion of pectin and cellulose (Macquet et al., 2007). Cellulose synthesized by CELLULOSE SYNTHASE 5 (CESA5), CESA3, and COBRA-LIKE 2 (COBL2) is required for mucilage adherence through xylans synthesized by MUCILAGE-MODIFIED 5/MUCILAGE RELATED 21 (MUM5/MUCI21), IRREGULAR XYLEM 7 (IRX7), and IRX14 and pectins (Mendu et al., 2011; Sullivan et al., 2011; Ben-Tov et al., 2015; Griffiths et al., 2015; Voiniciuc et al., 2015b; Hu et al., 2016a,b; Ralet et al., 2016). *SOS5* and *FEI2* mutants have a reduction in pectic-mucilage adherence similar to *cesa5*, yet how these two genes mediate adherence is still unknown (Harpaz-Saad et al., 2011; Griffiths et al., 2014).

When visualizing cellulose in the adherent mucilage layer, the distribution of cellulose can be divided into two distinct regions; an intensely staining ray-like structure located above the central columella of each cell, and a diffuse staining region between the rays (Macquet et al., 2007; Griffiths et al., 2014, 2015). Mutation of *cesa5, mum5*, and *cobl2* results in a loss of mucilage adherence and staining of regions of diffuse cellulose in the adherent layer is lost while cellulose observed in rays appears to be mostly intact (Mendu et al., 2011; Sullivan et al., 2011; Ben-Tov et al., 2015; Voiniciuc et al., 2015b; Ralet et al., 2016). Although *sos5* and *fei2* mutants have a similar loss of pectin-mucilage adherence, they have an inverse effect on cellulose structure within the adherent layer; the ray structure is completely absent yet the diffuse staining region remains (Harpaz-Saad et al., 2011; Griffiths et al., 2014). The phenotypes of *cesa5* and *sos5* seeds are clearly different and a *cesa5 sos5* double mutant has a more severe phenotype than either single mutant, demonstrating that these two genes function independently to mediate mucilage adherence (Griffiths et al., 2014).

While the mechanism behind the attachment of mucilage pectin to CESA5 synthesized cellulose requires xylan branches on RG-I synthesized by MUM5 (Ralet et al., 2016), the mechanism underlying SOS5 and FEI2 mediated mucilage adherence remains unresolved (Griffiths et al., 2014). Here, we further investigate the role of SOS5 and FEI2 in mucilage adherence using a combination of genetics, polysaccharide chemistry, and localization of the SOS5 protein. We define two unique genetic pathways that mediate mucilage adherence, one through cellulose and another through SOS5-FEI2.

## Materials and Methods

### Plant Material

The mutants *cesa5-2* (SALK_099008), *mum5-2* (WiscDSLox line 503F10), and *sos5-2* (SALK_125874) are in the Col-0 accession (Xu et al., 2008; Sullivan et al., 2011; Ralet et al., 2016). The T-DNA insertion line SAIL 150_A08, in the Col-0 accession, was identified in the SIGnAL database (Alonso et al., 2003^1^), seeds were obtained from the Nottingham *Arabidopsis* Stock Centre^2^ and a homozygous line was identified which we named *fei2-3*. Double mutants for *cesa5-2 fei2-3*, *mum5-2 cesa5-2*, *sos5-2 mum5-2*, and *sos5-2 fei2-3* were identified from F2 plants generated from crosses between the two respective single mutants by PCR using the primers listed in Supplementary Table 1, in combination with the insert specific primer LBb1.3 for SALK lines, and SAIL LB1 for SAIL lines (**Table 1**). Seeds were produced in a glasshouse (18–28°C), with a minimum photoperiod of 13 h provided by supplemental lighting. Plants were grown in compost (Tref substrates) and watered with Plant-Prod nutritive solution (Fertil^3^). As the seed coat is a maternal tissue the F1 and F2 seed mucilage phenotypes were determined using the F2 and F3 seed, respectively. For all comparisons, seed lots used were obtained from plants that had been simultaneously cultivated and harvested.

**Table 1:** Sugar composition of adherent mucilage from wild-type, *cesa5-2*, *sos5-2*, and *fei2-3* extracted with RGase, following water extraction.

	Wild-type	*cesa5-2*	*sos5-2*	*fei2-3*
Rha	3.08 (0.17)	1.41 (0.14)*	1.38 (0.09)*	1.37 (0.08)*
Fuc	0.07 (0.01)	0.06 (0.004)	0.10 (0.03)	0.10 (0.05)
Ara	0.16 (0.009)	0.17 (0.02)	0.15 (0.009)	0.19 (0.02)
Xyl	0.10 (0.01)	0.09 (0.01)	0.09 (0.01)	0.14 (0.08)
Man	0.21 (0.01)	0.24 (0.02)	0.16 (0.02)	0.22 (0.01)
Gal	1.41 (0.05)	1.47 (0.06)	1.07 (0.31)	1.67 (0.13)*
Glu	6.51 (0.18)	6.48 (0.14)	5.93 (0.20)	7.96 (0.26)*

*Values in parentheses are SE of three independently extracted samples from three biological repeats. Asterisks indicate a significant difference from wild-type (Mann–Whitney *U* test, p < 0.001).*

### Cloning of pro*UBQ10*:*SOS5*-mCitrine

The pro*UBQ10:SOS5-mCitrine* construct is based on pGREEN179 (GenBank: EUO48866.1; Hellens et al., 2000) plant transformation vector and contains PCR fragments of the 611 bp promoter region including the 5′ UTR of UBQ10 (At4g05320), the 27 amino acid (aa) residue secretion signal of SOS5/FLA4, a SRVPV linker, mCitrine (Shaner et al., 2005; GenBank : AEJ82308.1; aa residues 1–239), followed by most of the SOS5/FLA4 coding region (At3g46550; aa residues 29–420) and the HSP18.2 (At5g59720) terminator (Nagaya et al., 2010). All primers used for cloning are shown in Supplementary Table 1.

### Cytochemical Staining and Immunolabeling Procedures

Ruthenium red staining was performed as previously described (Sullivan et al., 2011). For cellulose staining, seeds were imbibed and shaken in water for 1 h, followed by staining for 1 h with 0.01% (w/v) Direct Red 23 (DR; Sigma–Aldrich) in a 100 mM NaCl solution (pH 5.0). Seeds were rinsed then imaged on a Leica SP5 II AOBS confocal microscope with a Tandem HyD detector (561 nm excitation and 570–660 nm emission). Whole mount immunolabeling procedures were performed as described in Voiniciuc et al. (2015a) with LM14 and LM21 antibodies^4^ then imaged on a Leica SP5 II AOBS confocal microscope with a Tandem HyD detector (488 nm excitation and 500–550 nm emission for AlexaFluor 488 and 561 nm excitation and 570–660 nm emission for DR).

For imaging birefringence of polarized light, seeds were imbibed in water for 20 min, mounted on a glass slide and observed using a Nikon SMZ800 binocular loop, a Nikon Coolpix E4500 camera and a Nikon simple polarized light adaptor (Nikon MNN40920^5^).

Seeds expressing pro*UBQ10:SOS5-mCitrine* were excised from developing siliques at approximately 10 days post-anthesis (dpa). Seeds were stained with 50 μM FM4-64 (Invitrogen) for 0.5 h then imaged on a Leica SP5 II AOBS Tandem HyD microscope with an excitation/emission of 516/525–575 nm for mCitrine, and 516/600–700 nm for FM4-64. All images were processed using ImageJ version 1.47v (Freeware, National Institutes of Health, USA^6^).

### Extraction and Analysis of Mucilage

Analysis of the monosaccharide composition of mucilage was performed as detailed in Sullivan et al. (2011). Briefly, 200 mg of seeds were hydrated in 4 mL of distilled water and shaken head-over-tail for 3 h. The supernatants were collected and filtered through a disposable glass microfiber filter (13 mm diameter, 2.7 μm pore size; Whatman^7^). Seeds were then rinsed twice, and rinses were discarded. The adherent mucilage layer was digested by adding 0.1 nkat of rhamnogalacturonan hydrolase (Swiss-Prot Q00018) provided by Novozymes^8^ in 4 mL of 50 mM sodium acetate, pH 4.5. Seeds were incubated with the enzyme for 16 h at 40°C, then centrifuged (8000 × *g*, 3 min), and the supernatant was collected and filtered through a disposable glass microfiber filter. Seeds were washed again three times with 5 mL of 50 mM sodium acetate buffer, pH 4.5, then treated with 0.9 nkat of Maxazyme^®^ cellulase (DSM, Seclin, France) at 40°C for 16 h, before centrifuging (8000 × *g*, 3 min).

Total uronic acid (GalA) and neutral sugar contents were determined with the automated *m*-hydroxybiphenyl and orcinol methods, respectively (Thibault, 1979; Tollier and Robin, 1979). Individual neutral sugars were analyzed as alditol acetate derivatives (Blakeney et al., 1983) by gas–liquid chromatography (Perkin Elmer Gas chromatograph) after hydrolysis with 2 M trifluoroacetic acid at 121°C for 2.5 h. Sugar amounts from different mutant lines were compared using the Mann–Whitney *U* test to determine statistically significant differences.

For HP-SEC analysis, 200 mg of seeds were hydrated in 4 mL of distilled water, and shaken head-over-tail for 3 h. The supernatant was extracted following centrifugation (8000 × *g*, 3 min) filtered through a disposable glass microfiber filter, and boiled for 5 min. HP-SEC analysis of water-extracted mucilage was performed at room temperature with a Shodex OH SB-G precolumn and a Shodex OH-Pack SB-805 HQ column^9^. Elution was carried out with 50 mM sodium nitrate buffer at a constant flow rate of 60 mL h^-1^. Polymers were detected with a differential refractometer (VE 3580 RI detector) and a Viscotek 270 Dual Detector (dual laser light scattering, wavelength = 670 nm, 90° and 7° combined with a differential pressure viscometer (Malvern Instruments^10^). Detectors were calibrated with a pullulan standard with a narrow molecular mass distribution (*M*w = 145 618 D, *M*_n_ = 139 180 D, [η] = 54 mL g^-1^ at 30°C in 0.1 M sodium nitrate, dn/dc = 0.147 mL g^-1^; Malvern Instruments). Samples were automatically injected through a 50 μL loop. Data analysis was performed using OmniSec version 4.5 software (Malvern Instruments).

## Results

### Both FEI2 and SOS5 Mediate Mucilage Adherence through a Common Pathway that Is Independent of Cellulose

**FIGURE 1:**
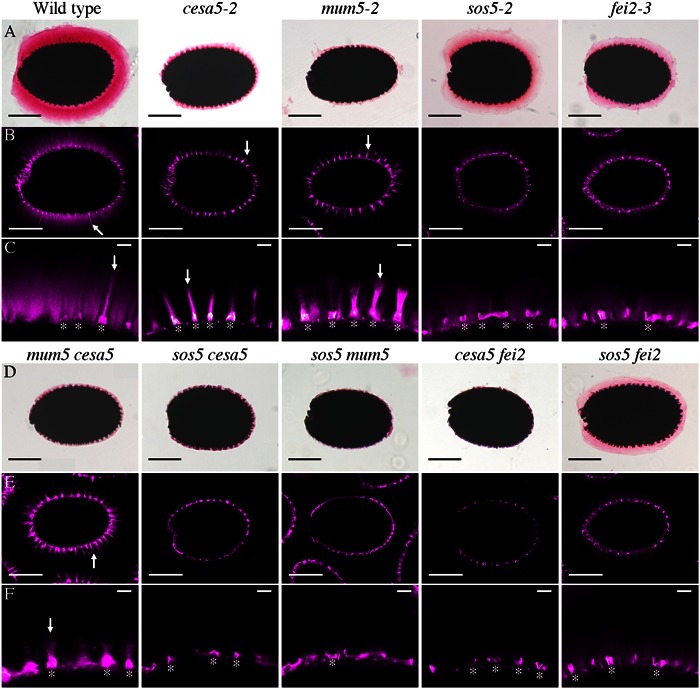
**Two distinct pathways regulate mucilage adherence to the seed coat, one is mediated by CESA5 and MUM5 and the other by SOS5 and FEI2. (A,D)** Adherent mucilage from seeds of the indicated genotypes was stained with ruthenium red for pectin, bars = 200 μm. **(B,C,E,F)** seeds stained with direct red for cellulose, bars = 200 μm **(B,E)**, or 25 μm **(C,F)**. Arrows indicate rays. Asterisks indicate the location of the columella.

Cellulose synthase 5 and SOS5 have previously been shown to mediate mucilage adherence through two distinct and independent mechanisms (Griffiths et al., 2014). In order to clarify the relationship between CESA5, SOS5, and other proteins involved in mucilage adherence, the pectin and cellulose that form the adherent mucilage were examined in single and double mutants. Ruthenium red (RR) stained mutant seeds of *cesa5-2, sos5-2, mum5-2*, and a new mutant allele of FEI2, *fei2-3* (SAIL 150_A08), were compared to wild-type. All four had little RR staining of pectin as previously described (**Figure 1A**; Harpaz-Saad et al., 2011; Mendu et al., 2011; Sullivan et al., 2011; Griffiths et al., 2015; Voiniciuc et al., 2015b; Ralet et al., 2016). Nevertheless, *sos5-2* and *fei2-3* seeds often had larger unstained halos surrounding seeds, which was rarely observed for *cesa5-2* or *mum5-2* seeds (**Figure 1**). When stained for cellulose with direct red (DR), two regions can be distinguished in wild-type mucilage, a central ray region (see arrow, **Figure 1**) above the columella (see asterisks, **Figure 1**), and a diffuse staining region around the rays. While *cesa5-2* and *mum5-2* seeds still had observable ray structures (see arrows, **Figures 1B,C**), these were no longer present in *sos5-2* and *fei2-3* mucilage. The latter mutants still had diffusely stained cellulose, whilst this was absent in *cesa5* and *mum5* (**Figures 1B,C**). We can therefore expand on the previously elucidated independent mechanisms for mucilage attachment by CESA5 and SOS5 (Griffiths et al., 2014) to define two distinct pathways mediating pectin-mucilage adherence based on cellulose staining. First, MUM5 is involved in the pathway required for adherence through cellulose synthesized by CESA5, which is observed as a diffuse staining region within mucilage. A second pathway requires FEI2 and SOS5 for the formation of cellulosic rays. These two pathways are clearly delineated by the staining patterns of cellulose in the adherent mucilage layer.

To confirm these pathways genetically, double mutants between the different components mediating mucilage adherence were generated and examined. The RR stained halo of mucilage observed in the *cesa5-2 mum5-2* double mutant was similar to *cesa5-2* and *mum5-2* single mutants (**Figure 1D**) and did not represent an additive phenotype. Furthermore, rays of cellulose were still visible in the *cesa5-2 mum5-2* double mutants (see arrow, **Figures 1E,F**) confirming that CESA5 and MUM5 are involved in a common mechanism that mediates mucilage adherence and the formation of the diffusely stained cellulose regions. In contrast, *sos5-2 mum5-2* seeds exhibited a greater reduction in pectin to that observed in the *mum5-2* and *sos5-2* single mutants and there was no staining of either rays or diffuse regions in double mutant mucilage (**Figures 1D–F**). This was similar to the additive phenotype previously reported for *cesa5-2 sos5-2* (**Figures 1D–F**), (Griffiths et al., 2014) in accord with SOS5 being involved in a mechanism independent of the attachment of mucilage pectin to cellulose through xylan. Similarly, a drastic reduction in RR staining and an absence of cellulose staining were observed for *cesa5-2 fei2-3* mucilage (**Figures 1D–F**) suggesting that FEI2 also affects mucilage adherence through a mechanism independent of CESA5. To determine whether this could be the same as that for SOS5 a *sos5-2 fei2-3* mutant was generated. The mucilage of double mutant seeds did not appear to be more severely affected than either single mutant, with diffuse staining of cellulose still observed in the adherent layer (**Figures 1D,E**). Taken together these results demonstrate that in the two independent pathways that mediate mucilage adherence, one requires cellulose synthesized by CESA5 and xylans synthesized by MUM5, and the second comprises SOS5 and FEI2.

**FIGURE 2:**
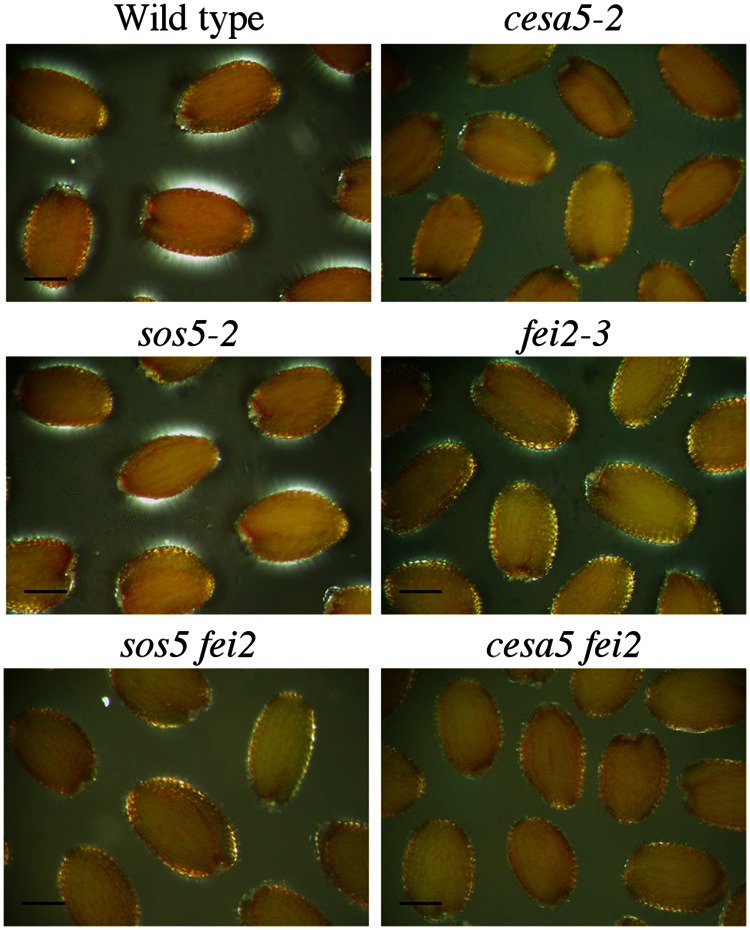
**Polarized light birefringence of crystalline cellulose microfibrils is not affected in *sos5-2* seeds.** Imbibed seeds were visualized under polarized light. Bars = 200 μm.

To further examine the role of SOS5 and FEI2 in cellulose mediated mucilage adherence, polarized light was used to examine crystalline cellulose in the adherent mucilage layer of *cesa5-2*, *sos5-2*, *fei2-3*, *sos5-2 fei2-3*, and *cesa5-2 fei2-3* seeds (**Figure 2**). When hydrated in water and viewed under polarized light, the mucilage of wild-type seeds was seen as a bright halo, while no reflection of polarized light was observed in *cesa5-2* mucilage (**Figure 2**). Interestingly, the adherent mucilage of both *sos5-2* and *fei2-3* seeds still displayed birefringent halos, although these were smaller and exhibited some curious differences. The reflection of light was reduced in *fei2-3* and *sos5-2 fei2-3* seeds compared to wild-type or *sos5-2* seeds (**Figure 2**). Finally, *cesa5-2 fei2-3* birefringent halos were similar to *cesa5-2* seeds, and reduced compared to *fei2-3* seeds, in agreement with FEI2 function being independent of CESA5. These results also suggest that *fei2-3* seeds have a more severe phenotype compared to *sos5-2* seeds, indicating a function for FEI2 independent of that of SOS5 that could be involved in determining the degree of cellulose crystallinity in adherent mucilage.

### Heteromannan Structuring of Cellulose Appears to Be Independent of the SOS5-FEI2 Pathway

**FIGURE 3:**
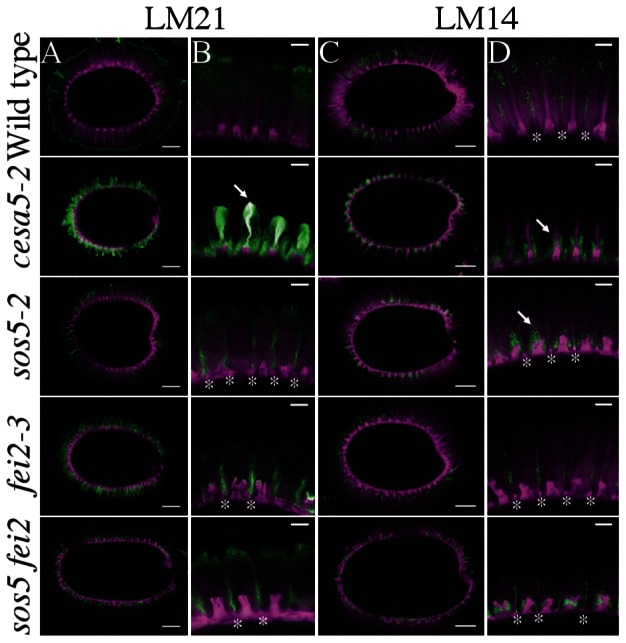
**Immunolabeling of *cesa5*, *fei2*, and *sos5* adherent mucilage with LM21 against heteromannans and LM14 against AGPs. (A,B)** LM21 antibody signal in green, cellulose stained with DR in magenta. **(C,D)** LM14 antibody signal in green, cellulose stained with DR in magenta. **(A,C)** Bars = 100 μm. **(B,D)** Bars = 25 μm. Arrows indicate rays, asterisks indicate radial walls.

Reductions in birefringence of crystalline cellulose have also been observed for other components involved in adherent mucilage structure, notably two genes that synthesize heteromannans, *CSLA2* and *MUCI10* (Yu et al., 2014; Voiniciuc et al., 2015a). Since FEI2 has a similar reduction in crystalline cellulose birefringence and also appears to function independently of CESA5, we investigated the relationship between CESA5, SOS5, and FEI2 with heteromannan through immunolabeling with the LM21 antibody (Marcus et al., 2010). The LM21 signal is relatively weak in wild-type seeds, but extensive labeling of the adherent mucilage halo can be observed, although rays are not strongly labeled by LM21 (**Figures 3A,B**; Voiniciuc et al., 2015b). In contrast *cesa5-2* seeds had a strong LM21 signal, which was much more intense than that of wild-type, and was also colocalized with rays indicating a link between production of cellulose and heteromannan (see arrow, **Figure 3B**). The localization of LM21 signal in *sos5-2*, *fei2-3*, and *sos5-2 fei2-3* mucilage was the inverse of that observed for *cesa5-2* seeds, with labeling being particularly intense in mucilage regions situated above the radial walls, corresponding to the interphase between the mucilage released from two independent epidermal cells (see asterisks, **Figure 3B**); the *sos5-2 fei2-3* double mutant was indistinguishable from either single mutant. The localization of LM21 signal in *sos5-2* and *fei2-3* mucilage was consistent with a general loss of mucilage adherence and faulty ray organization, indicating that SOS5 and FEI2 function independently of heteromannan in mediating mucilage adherence and cellulose crystallinity.

We also examined the localization of LM14, an antibody specific to arabinogalactan proteins (Moller et al., 2008) to try to understand the localization of SOS5, and the effects of CESA5 and FEI2 on SOS5 localization. In wild-type seeds, LM14 signal appeared to be more specific to the interphase between mucilage released from two different epidermal cells, and it did not label rays (**Figures 3C,D**). In *cesa5-2* mucilage, LM14 was specifically localized around the ray, and appears to coat the base of the ray, yet the signal did not colocalize with the DR stain of cellulose (see arrow, **Figure 3**). The localization of LM14 in *cesa5-2* seeds is very similar to that of *cesa5-1* seeds labeled with the RG-I specific antibody CCRC-M36 (Griffiths et al., 2014, 2015), which suggests an association between arabinogalactan proteins and RG-I, potentially serving to organize the ray. In *sos5-2* and *sos5 fei2* double mutant seeds, LM14 signal was localized around the columella, with some signal observable above the columella despite the lack of a cellulosic ray (see arrow, **Figure 3**). Similar to wild-type seeds, clear signals were observed in the interphase region of mucilage released from two different cells in *sos5-2*, *fei2-3*, and *sos5-2 fei2-3* seeds. The arabinogalactan proteins labeled by LM14 in the mucilage are not, therefore, uniquely SOS5, which suggests that other arabinogalactan proteins are involved in structuring adherent mucilage.

### FEI2 Has a Predominant Role in Mucilage Adhesion Compared to SOS5

**FIGURE 4:**
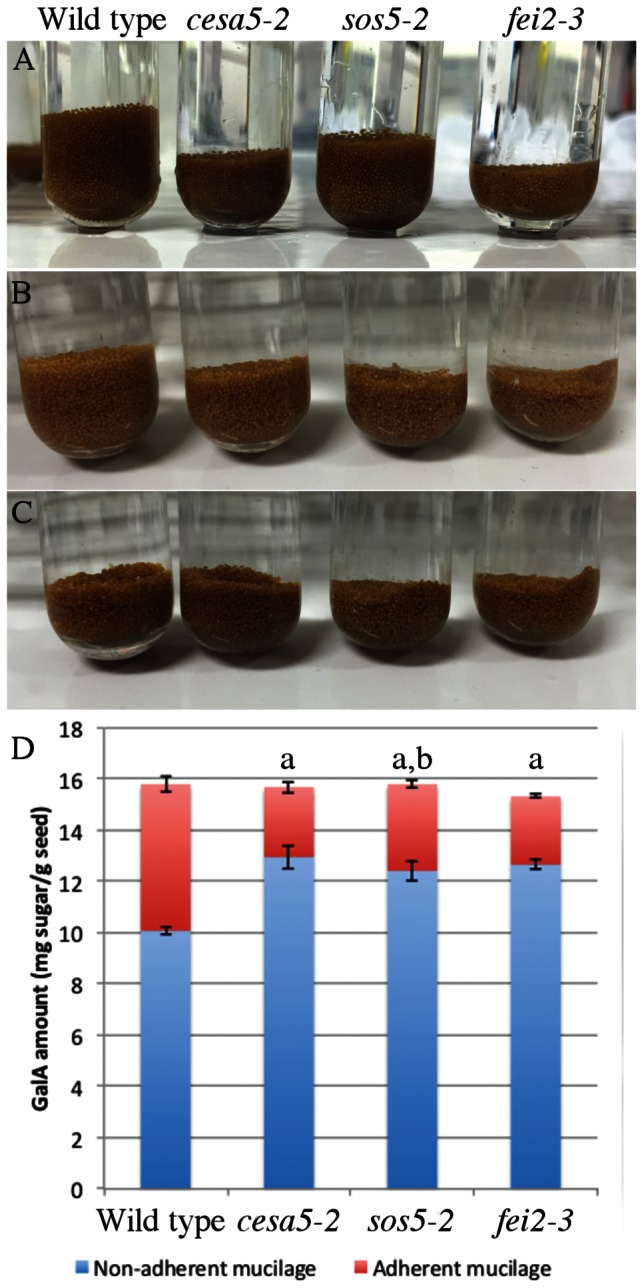
***sos5* seeds have more adherent mucilage than *fei2-3* and *cesa5-2*.** Images of seeds after sequential extraction of mucilage from 200 mg of seeds with **(A)** water for 3 h, **(B)** rhamnogalacturonan hydrolase (RGase) for 16 h, and **(C)** cellulase for 16 h. **(D)** Partitioning of galacturonic acid (GalA) between water and RGase extracts. Error bars represent SE of three independently extracted samples. The letter “a” indicates a significant difference from wild-type in non-adherent mucilage amounts. The letter “b” indicates a significant difference from *cesa5-2* and *fei2-3* seeds for the adherent mucilage fraction. Mann–Whitney *U* test, *P* < 0.05.

To gain more information concerning the mechanism by which SOS5 and FEI2 intervene in mucilage adherence a detailed analysis of adherent mucilage composition was carried out. Mucilage was extracted sequentially with water from the same weight of seeds for each genotype followed by digestion of the inner mucilage layer with rhamnogalacturonan hydrolase (RGase). Finally, cellulase was used to hydrolyse the cellulose still attached to the seed coat. Following the water extraction differences in the volume of the seeds and remaining adherent mucilage were observed compared to wild-type (**Figure 4A**). Wild-type seeds had the greatest volume, followed by *sos5-2* seeds, while *cesa5-2* and *fei2-3* seeds occupied the smallest volume (**Figure 4A**). Following RGase treatment, however, all three mutants had a similarly reduced volume compared to wild-type (**Figure 4B**). Finally, after cellulase treatment the seed volume for all four genotypes was equivalent (**Figure 4C**). The difference in seed volume for *sos5-2* and *fei2-3* seeds prior to RGase treatment again indicated that *fei2-3* has a stronger effect on mucilage adherence than *sos5-2*.

The total amounts of acid sugars extracted with water or following RGase digestion were quantified (**Figure 4D**), and the amounts of individual monosaccharides were determined in RGase hydrolysates (**Table 1**). Consistent with their loss of mucilage adherence phenotype, *cesa5-2*, *sos5-2*, and *fei2-3* seeds had increased amounts of sugars extracted from non-adherent mucilage, and reduced amounts from adherent mucilage, as previously reported (Harpaz-Saad et al., 2011; Mendu et al., 2011; Sullivan et al., 2011; Griffiths et al., 2014); amounts of Rha in RGase extracts were significantly lower in all three mutants compared to wild-type (**Table 1**; Mann–Whitney *U* test, *p* < 0.001). Interestingly, *sos5-2* seeds had more GalA in RGase hydrolysates than *cesa5-2* or *fei2-3* (**Figure 4D**; Mann–Whitney *U* test, *p* < 0.05), in accord with the larger volume of their seeds and adherent mucilage after water extraction alone (**Figure 4A**). This confirmed that *sos5-2* mutation has a weaker effect on mucilage adherence than defects in the other genes. Together with the images of birefringence, these results demonstrate that in addition to differences between CESA5 and the SOS5-FEI2 pathway in mediating adherence the overlap between the roles of SOS5 and FEI2 is not complete and suggest that FEI2 has additional functions beyond SOS5.

**FIGURE 5:**
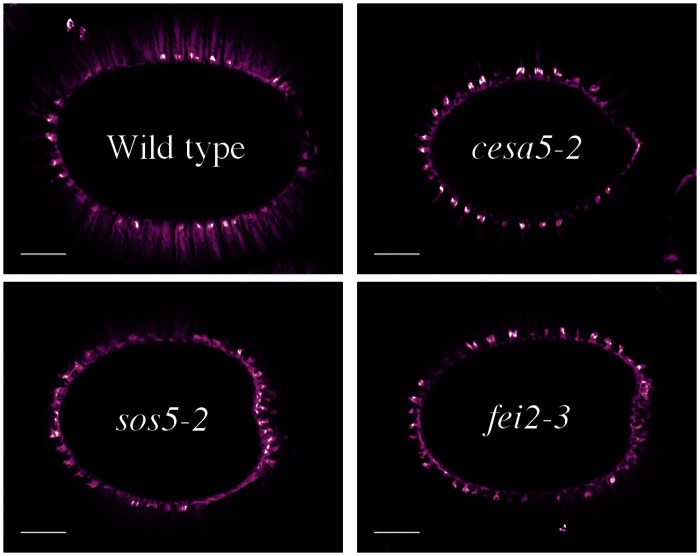
**Structure of cellulose in adherent mucilage after digestion with rhamnogalacturonan hydrolase for 16 h.** Cellulose was stained with direct red. Bar = 200 μm.

To examine whether the presence of RG-I is responsible for the organization of cellulose into two differently staining populations, seeds were stained with DR following RGase digestion. Brightly stained cellulose was still observed in the adherent mucilage of wild-type seeds as both rays and diffuse regions (**Figure 5**). Furthermore, no modification was observed in the structure of the cellulose observed in the mutants with *cesa5-2* mucilage still having cellulose in rays, while *sos5-2* and *fei2-3* cellulose still appeared diffuse (**Figure 5**). These results suggest that the presence of rays does not require RG-I, and that rays could be pre-determined in the mucilage pocket.

**FIGURE 6:**
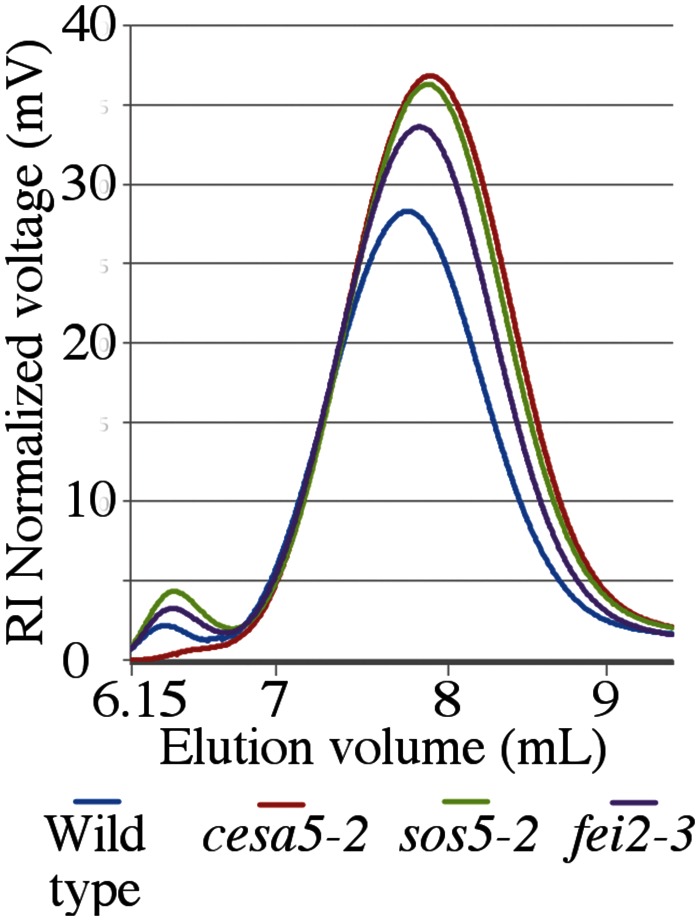
**Water-extracted mucilage from *sos5* and *fei2* has distinct macromolecular properties compared to wild-type and *cesa5*.** Water extracted mucilage was separated by size-exclusion chromatography combined with refractive index detection. Plots are the average value of three independent biological replicates.

The macromolecular characteristics of mucilage polysaccharides extracted with water from mutant seeds were examined using HP-SEC to obtain information about how SOS5 and FEI2 affect polysaccharide conformation and interactions. Wild-type mucilage separated into two distinct polymeric fractions, an initial small peak representing large molecular weight polymers, and a second larger peak representing relatively smaller molecular weight polymers (**Figure 6**; **Table 2**; Macquet et al., 2007). *cesa5* mutant seeds lacked the large molecular weight polymeric fraction (**Figure 6**; Sullivan et al., 2011). Comparable results have also been observed for *irx7*, *irx14*, and *mum5* (Macquet et al., 2007; Hu et al., 2016a,b). In contrast, both *sos5-2* and *fei2-3* extracts retained the first peak, showing that the larger polymeric aggregates are present in the mucilage of these mutants (**Figure 6**), and were more abundant in *sos5-2*, compared to wild-type. There was, however, a significant reduction in both the weight-average and the number-average molecular weights (*M*_w_ and *M*_n_, respectively) in *sos5-2* and *fei2-3* polymers, compared to wild-type (**Table 2**), the polydispersity index (*M*_w_/*M*_n_) being further from 1. This population of RG-I polymers in *sos5-2* and *fei2-3* mucilage had, therefore a greater range of sizes, and were on average smaller than in the wild-type. The intrinsic viscosity of *sos5-2* and *fei2-3* was also reduced compared to wild-type in this first polymeric fraction (**Table 2**), in accord with the reduced average polymer size. In contrast, the second polymeric fraction peaked at higher values in all three mutants compared to wild-type (**Figure 6**), with a significantly increased peak surface for *cesa5-2* and *sos5-2* (**Table 2**), in accord with the increased amount of mucilage extracted with water in these mutant seeds. In this second polymeric fraction polymers were shorter in the mutants compared to wild-type, being particularly marked for *cesa5-2*. Finally, *cesa5-2* and *sos5-2* had reduced intrinsic viscosity compared to wild-type mucilage. These results demonstrate that there are clear alterations to the macromolecular properties of the polysaccharides with a tendency toward smaller and likely shorter RG-I polymers in the mutants, which is most severe in *cesa5-2*. Furthermore, the mucilage polymers extracted with water from *sos5-2* and *fei2-3* seeds differ from those of *cesa5-2* as they retain the population of high average molecular weight. This difference in SEC profile is in agreement with SOS5 and FEI2 having a function that is largely independent of cellulose.

**Table 2:** Macromolecular characteristics of wild-type Col-0, *cesa5-2*, *sos5-2*, and *fei2-3* water-extracted mucilage.

	RI Area	M_w_ (kD)	M_n_ (kD)	M_w_/M_n_	[η] (dL/g)
**First Polymeric Fraction**					
Wild-type	0.8425 (0.13)	22717 (826)	22325 (951)	1.02 (0.007)	7.88 (0.14)
*sos5-2*	**2.16 (0.13)**	**18415 (600)**	**16535 (886)**	**1.12 (0.027)**	**6.90 (0.15)**
*fei2-3*	1.61 (0.29)	**17253 (840)**	**15540 (1156)**	**1.11 (0.029)**	**7.24 (0.06)**
**Second Polymeric Fraction**					
Wild-type	32.695 (0.76)	581 (7.5)	554 (6.9)	1.05 (0.002)	5.96 (0.08)
*cesa5-2*	**44.39 (2.55)**	**429 (8.7)**	**415 (9.3)**	1.03 (0.003)*	**5.79 (0.06)**
*sos5-2*	**41.99 (1.52)**	569 (5.1)	537 (5.6)	1.06 (0.06)	5.71 (0.06)*
*fei2-3*	38.26 (4.75)	**541 (5.0)**	**519 (6.7)**	1.04 (0.004)	5.91 (0.07)

*Values in parentheses are SE values of three biological replicates. M_w_, weight-average molar mass; M_n_, number-average molecular weight, M_w_/M_n_, polydispersity index, [η], number-average intrinsic viscosity. Asterisk indicates a significant different value from wild-type *p* < 0.05, bold values indicate a significantly different value from wild-type *p* < 0.001; Mann–Whitney *U* test.*

### SOS5 Is Localized throughout the Mucilage Pocket

**FIGURE 7:**
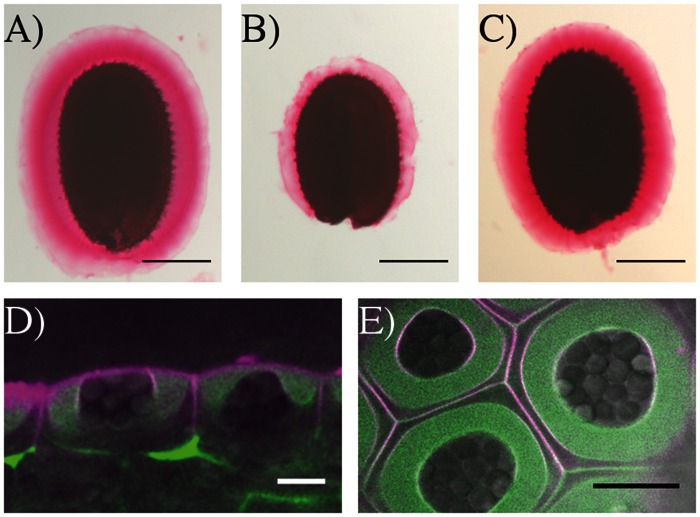
**SOS5-mCitrine is localized throughout the mucilage pocket and on the plasma membrane.** Ruthenium red pectin in the adherent mucilage of **(A)** wild-type **(B)** *sos5-2* and **(C)** pro*UBQ10:mCitrine-SOS5 sos5-2* seeds. Optical cross-sections **(D)** side view and **(E)** top view of SOS5-mCitrine (green) counter stained with the plasma membrane dye FM4-64 (magenta) and overlaid with transmitted light (gray) in the epidermal cells of 10 DAP developing seeds. Bars 200 μm **(A–C)** or 10 μm **(D,E)**.

In roots the SOS5-FEI2 pathway has been proposed to intervene in cell wall sensing and the initiation of a signaling cascade involving hormones. The SOS5 protein is an extracellular protein and has a GPI domain that could anchor it to the plasma membrane. To gain further insight into the potential mechanism through which SOS5 acts, localization of the SOS5 protein was examined using a reporter protein. *SOS5* was cloned under the control of the *UBIQUITIN 10* promoter, in a translational fusion with the mCitrine fluorescent protein. pro*UBQ10-SOS5-mCitrine* plants fully complemented the *sos5-2* mucilage adhesion phenotype, indicating that this fusion protein is functional (**Figures 7A–C**). Next we examined the localization of this protein in developing seeds at approximately 10 days post-anthesis (DPA), when mucilage polysaccharides are being actively secreted into the apoplast of seed coat epidermal cells. While we cannot eliminate the possibility that the mCitrine molecule is cleaved from SOS5, the SOS5-mCitrine signal was predominantly detected with uniform labeling throughout the polysaccharides present in the mucilage pocket (**Figures 7D,E**). SOS5-mCitrine was also observed at the plasma membrane, as demonstrated by colocalization with the plasma membrane dye FM4-64. These results confirm the predicted SOS5 plasma membrane localization and previous observations of SOS5 being localized to the plasma membrane (Shi et al., 2003), and also show that in seed coat epidermal cells the majority of the protein is localized in the apoplast where it would be associated with mucilage polysaccharides.

## Discussion

In *Arabidopsis*, seed mucilage forms two discrete layers and studies of how these are formed are providing valuable information concerning our basic understanding of polysaccharide interactions. Here, we expand our knowledge of the mechanisms involved in mucilage adherence and further define two unique pathways that mediate polysaccharide interactions. Based on mutant mucilage phenotypes and double mutant analysis, CESA5 and MUM5 define one pathway that requires cellulose and hemicelluloses for mucilage adherence (**Figure 1**). A second pathway involving SOS5 and FEI2 functions independently of the CESA5-MUM5 pathway to mediate adherence. Single mutants can be divided into two distinct classes based on cellulose staining, one comprising *cesa5* and *mum5* where rays of cellulose are intact yet diffuse staining cellulose is lost, and a second class including *sos5* and *fei2* where rays fail to form, while diffusely stained cellulose remains (**Figure 1**). We show that neither SOS5 nor FEI2 play a significant role in crystalline cellulose formation within mucilage (**Figure 2**). Finally, despite the similarities in the phenotypes of *sos5* and *fei2* seeds, we identify some clear differences in mutant phenotypes that suggest that FEI2 has a role in cell wall organization beyond that mediated by SOS5 (**Figures 4–6**).

### Two Distinct Pathways Mediate Mucilage Adherence

**FIGURE 8:**
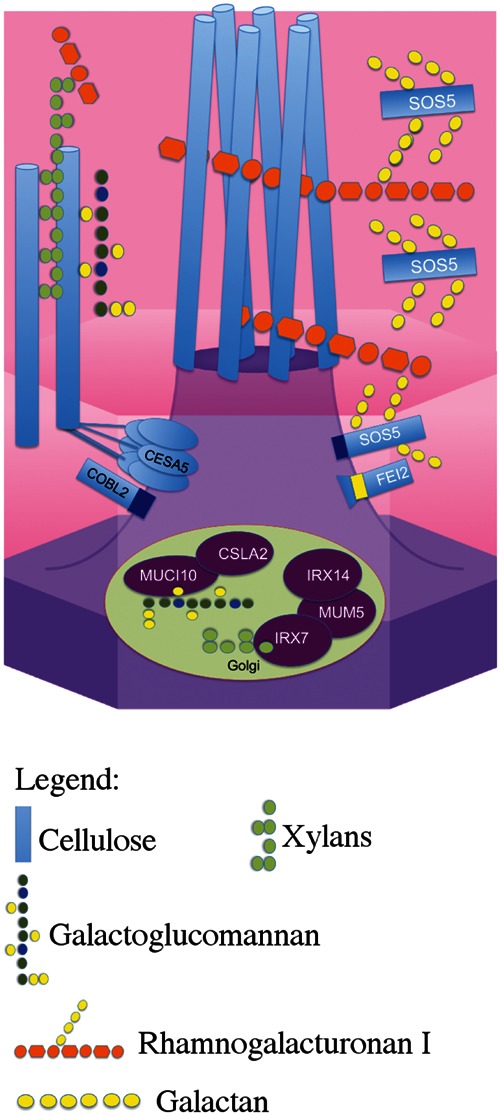
**Schematic model of the two distinct pathways that mediate mucilage adherence.** CESA5 synthesizes cellulose which is connected to xylan branches synthesized by MUM5, IRX14, and IRX7 (Voiniciuc et al., 2015b; Hu et al., 2016a,b). Xylans are directly linked to pectin RG-I and can bind it to cellulose (Ralet et al., 2016). A second independent pathway mediating adherence requires SOS5 and FEI2 and is specifically required for ray formation, potentially through RG-I (Griffiths et al., 2014, this study).

Previously, CESA5 and SOS5 had been shown to function independently in mucilage adherence (Griffiths et al., 2014). Here, we have expanded this analysis to include other genes that affect mucilage partitioning into layers, and define two distinct pathways that control adherence (**Figure 8**). The first pathway involves cellulose that is connected to the major component of mucilage, the pectin RG-I, through xylan branches synthesized by MUM5/MUCI21 (Harpaz-Saad et al., 2011; Mendu et al., 2011; Sullivan et al., 2011; Voiniciuc et al., 2015b; Ralet et al., 2016). Mutations in both of these genes produce seeds with similarly reduced mucilage adherence associated with the loss of the diffusely stained cellulose, while rays of cellulose remain above the columella (**Figure 1**), (Mendu et al., 2011; Sullivan et al., 2011; Voiniciuc et al., 2015b; Ralet et al., 2016). Furthermore, seeds from the *cesa5 mum5* double mutant did not show an additive phenotype confirming that the RG-I is attached through xylan branches to CESA5 synthesized cellulose. Furthermore, water-extracted mucilage from both single mutants does not contain the fraction of high molecular weight polymers, indicating that cellulose and hemicelluloses are required for the formation of these polymer aggregates in mucilage (**Figure 6**; **Table 2**), (Sullivan et al., 2011; Ralet et al., 2016). This pathway is also likely to involve IRX7 and IRX14, which are required for xylan biosynthesis and mucilage adherence, CESA3, and COBRA-LIKE 2 (COBL2) as *cobl2* seeds have similarly reduced mucilage adherence while cellulose rays are still present (Ben-Tov et al., 2015; Griffiths et al., 2015; Voiniciuc et al., 2015b; Hu et al., 2016a,b).

The second pathway requires both SOS5 and FEI2, and affects mucilage adherence independently of CESA5 and MUM5/MUCI21. SOS5 appears to have lesser affects on mucilage adherence and cellulose crystallinity compared to FEI2 (**Figures 1, 3**, and **4**), (Griffiths et al., 2014). The modest reduction in birefringence by *sos5-2* mucilage was coherent with the weaker effect on mucilage partitioning between inner and outer mucilage layers, but was unexpected given that cellulose staining itself appeared to be reduced in a similar manner to that of *fei2-3* mucilage. A *sos5 mum5* double mutant showed a similar additive phenotype to that previously described for *cesa5 sos5*, as did a *cesa5 fei2* double mutant (**Figures 1** and **2**), (Griffiths et al., 2014) demonstrating that neither SOS5 nor FEI2 affect mucilage adherence through the pathway involving RG-I attachment to cellulose through xylan synthesized by MUM5 (**Figure 1**). Instead, SOS5 and FEI2 appear to be required for the adherence of mucilage through a mechanism that also involves the organization of cellulose into rays.

While these two independent pathways are clearly defined, we know far more about the role of cellulose in mediating adherence than we do of the role of SOS5-FEI2. It is curious that as many as six unique genes have been implicated in cellulose mediated mucilage adherence, while only SOS5 and FEI2 function have been identified in the alternative pathway. Other CESAs must be involved in the synthesis of the ray component of mucilage, and it is still possible that SOS5 or FEI2 could interact with other CESAs. Interestingly, heteromannans synthesized by CSLA2 and MUCI10 do not appear to fit into either pathway as LM21 labeling was still observed within *cesa5*, *fei2*, and *sos5* mucilage (**Figure 3**). Furthermore, there is more pectin than wild-type in the adherent mucilage layer of *csla2* and *muci10* seeds (Yu et al., 2014; Voiniciuc et al., 2015a). The increased labeling of *cesa5* mucilage with LM21 is most likely due to increased accessibility to LM21 epitopes, potentially because there is less cellulose for heteromannans to bind (**Figure 3**). LM14 immunolabeling of adherent mucilage suggests that other arabinogalactan proteins are involved in ray organization and mucilage adherence in addition to SOS5. Further genetic analysis using double mutant combinations of *cesa5*, *sos5*, or *fei2* with *csla2, muci10* or other arabinogalactan proteins expressed in the seed coat would help to further define these two pathways mediating mucilage adherence.

### Function of SOS5

Salt-overly sensitive 5 was initially hypothesized to mediate cell adhesion through pectin in the cell wall as the fasciclin domains it contains are found in animal cell adhesion proteins (Shi et al., 2003). Subsequently, SOS5 was proposed to mediate cellulose biosynthesis through CESA5 due to the similar reduction in adherent mucilage observed in mutants affected in these two genes (Harpaz-Saad et al., 2011). This has since been negated, as *cesa5* and *sos5* have clearly different phenotypes with cellulose production apparently unaffected in *sos5* (Griffiths et al., 2014). More recently, SOS5 was proposed to function in signaling pathways that mediate cell expansion (Seifert et al., 2014; Xue and Seifert, 2015). Determining the precise mechanism of SOS5 function is, therefore, proving to be difficult. Here, we confirm that cellulose biosynthesis is largely unaffected in *sos5* mucilage (**Figure 2**). Additionally, we have identified a key difference between *sos5* and both *cesa5* and *fei2* as the adherent mucilage layer of *sos5* seeds contains more polysaccharide than either of the other mutants (**Figures 4A,D**). This increase in adherent mucilage is completely abolished on RGase treatment, indicating that it is mainly due to the amount of pectin, suggesting that SOS5 plays a relatively minor role in RG-I adherence compared to CESA5 and FEI2 (**Figure 4**). Finally, we have demonstrated that SOS5 is localized throughout the mucilage pocket as well as the plasma membrane in seed coat epidermal cells (**Figure 7**) suggesting that SOS5 may function at least partly in the apoplast in accord with a principal role in mediating pectin organization rather than cellulose biosynthesis or hormonal signaling. A structural role for SOS5 in organizing pectin is also supported by the modifications observed in the macromolecular properties of water-extracted mucilage polymers in *sos5*. Although *sos5* seeds have a similar composition to wild-type seeds, the polymers that form *sos5* mucilage were smaller in both polymeric fractions obtained by SEC. Furthermore, the polymers in *sos5* water-extracted mucilage were significantly different from *cesa5* mucilage, as they still contained the first very high-molecular weight population. This implicates SOS5 in the interactions between polysaccharides in mucilage, albeit independently of cellulose synthesized by CESA5 and hemicellulose synthesized by MUM5 and IRX14. Xylan has been shown to constitute a major connection between cellulose and mucilage RG-I (Ralet et al., 2016), while mutant phenotypes (**Figure 4**) indicate that SOS5 plays a lesser role in connecting these components. SOS5 could potentially organize the adherent mucilage halo through galactoglucomannans, which also play a role in mucilage organization and cellulose distribution (Voiniciuc et al., 2015a). Immunolabeling of heteromannans did not, however, reveal modifications to their distribution in the absence of SOS5, nor major changes in the intensity of their labeling (**Figure 3**) indicating that SOS5 is not required for their interactions within mucilage.

While the rays of cellulose are involved in mucilage expansion (Griffiths et al., 2015), how these are organized in the mucilage pocket prior to expansion remains to be determined. SOS5 could indirectly contribute to the orientation of cellulose microfibrils in the apoplast, or in the connections between cellulose and pectin. Other arabinogalactan proteins have been shown to be directly connected to pectin and hemicelluloses, forming a complex network (Tan et al., 2013), and we favor a similar hypothesis for the function of SOS5. Nevertheless, as indicated above the characteristics of water-extracted mucilage polymers from *sos5*, *cesa5*, and *mum5* (**Figure 5**), and other mutant phenotypes exclude SOS5 from acting via cellulose, either directly or indirectly through hemicelluloses. While we cannot exclude the possibility that SOS5 binds directly to cellulose or glucans in the ray structure, given all the evidence, we propose that SOS5 is involved in organizing the ray through a few key connections between RG-I polysaccharides, and potentially organizing the cell wall through specific localization or anchoring of pectin components. Loss of SOS5 therefore results in fewer connections between pectin, and slightly reduced polymer size, but also loss of cell wall organization and ray formation. Galactan side chains are required for SOS5 function (Basu et al., 2015), and determining what role they play in mucilage adherence and cell wall organization, and if the FAS protein–protein interaction domains are important for this role are important questions. Characterizing the glycosyl side chain composition of SOS5, examining the binding affinity of SOS5 to cell wall polymers and testing potential protein–protein interactions with FEI2 or other factors should help to clarify its function.

### Function of FEI2

FEI2 was proposed to control cellulose biosynthesis with this function being redundant with FEI1 in roots (Xu et al., 2008). Multiple reports have also implicated FEI2 as a key component of a signaling pathway that can monitor cell wall conditions, and initiate a cytosolic response, specifically altering cellulose biosynthesis (Xu et al., 2008; Steinwand et al., 2014; Basu et al., 2016). Yet the evidence supporting a role for FEI2 in cellulose biosynthesis is not convincing. Both *cesa6^prc1^* and *cobra1* (*cob1*) mutants can enhance the root phenotype of *fei1 fei2* mutants, suggesting that FEI1 and FEI2 function independently of these key components of cellulose biosynthesis (Xu et al., 2008). Here, we show that a *fei2 cesa5* double mutant is more severe than either single mutant, similar to the *cesa5 sos5* double mutant (**Figure 1**), (Griffiths et al., 2014), again suggesting that FEI2 functions independently of cellulose biosynthesis. The only evidence that FEI2 is involved in cellulose biosynthesis is the reduced amount of carbon incorporated into roots that have expansion defects (Xu et al., 2008). This could be a pleiotropic effect due to the inability of cells to expand, and not directly linked to cellulose biosynthesis (Xu et al., 2008).

Here, comparison of the phenotypes of *sos5* and *fei2* mucilage indicated that *sos5* phenotypes are weaker than those of *fei2* (**Tables 1** and **2**; **Figure 4**). Nonetheless, genetic analysis clearly supports a common pathway for mucilage organization involving SOS5 and FEI2 (**Figure 1**). This implies that FEI2 has some additional functions to those involving SOS5. FEI2 mutation had a stronger effect on crystalline cellulose amounts in the adherent mucilage layer, yet this reduction was not as severe as that observed for *cesa5* and is independent of *cesa5.* FEI2 has previously been localized to the plasma membrane (Xu et al., 2008) and the localization observed for SOS5 suggests a common function at the plasma membrane.

Both SOS5 and FEI2 have been linked to hormone signaling, either through auxin, ethylene, or abscisic acid (Xu et al., 2008; Seifert et al., 2014; Steinwand et al., 2014; Xue and Seifert, 2015). A role in cell wall recognition and signaling remain the primary hypothesis explaining the function of FEI2, although the modified mucilage partitioning observed in *sos5* and *fei2* is unconditional, unlike in roots where high salt concentrations are required for the phenotype to be observed. Despite this, both *sos5* and *fei1 fei2* can enhance the root growth phenotype of untreated *cob1* and *prc1* mutants, suggesting this phenotype is not conditional (Xu et al., 2008). These results argue against a role for either SOS5 or FEI2 in signaling, especially in mucilage as the phenotypes are manifested in the absence of salt treatments. IAA-ALANINE RESISTANT 4 (IAR4) is involved in auxin signaling and maintaining auxin homeostasis, and *iar4* mutants can restore the *fei1 fei2* root phenotype (LeClere et al., 2004; Steinwand et al., 2014). Nonetheless, *iar4* cannot restore the mucilage defect of *fei2* (Steinwand et al., 2014). We propose that SOS5 is required for interactions between cell wall components, which entail its correct localization within the apoplast, and that instead of a hormone-specific role, FEI2 would localize SOS5 in discreet regions in the plasma membrane. This hypothesis is consistent with the observation that SOS5 and FEI2 are required for the organization of cellulose into rays within mucilage and do not affect cellulose biosynthesis *per se* (Griffiths et al., 2014). FEI2 was previously proposed to localize cell wall biosynthetic machinery to a particular area of the plasma membrane (Xu et al., 2008), and as part of this mechanism, we hypothesize that this localization is vital for the function of SOS5.

In summary, here we further define two distinct pathways mediating mucilage adherence, one controlled by CESA5/MUM5, and another controlled by SOS5/FEI2. The *cesa5 mum5* phenotype demonstrates genetically that cellulose synthesized by CESA5 and xylans synthesized by MUM5 interact to mediate RG-I mucilage adherence, consistent with biochemical data (Voiniciuc et al., 2015a; Ralet et al., 2016). We have identified additional differences between the mutant phenotypes of *cesa5* and *sos5*/*fei2*, most notably the presence of crystalline cellulose in the adherent mucilage halo of *sos5*, and large molecular weight polymers present in *sos5* and *fei2* mucilage that are absent from *cesa5* mucilage. Finally, we have highlighted some minor differences in the phenotypes of *sos5* and *fei2* seeds, suggesting additional functions for FEI2 beyond the SOS5-FEI2 pathway. We demonstrate that SOS5 is localized throughout the mucilage pocket, consistent with a role of SOS5 in organizing the pectin component of mucilage around cellulose microfibrils that are required for ray formation.

## Author Contributions

JG and HN conceived experiments. JG, M-JC, HN, M-CR, and GS performed the experiments JG, M-CR, and HN analyzed the data, JG wrote and HN revised the manuscript with critical reading from M-CR and GS.

## Conflict of Interest Statement

The authors declare that the research was conducted in the absence of any commercial or financial relationships that could be construed as a potential conflict of interest.

## Footnotes

1 http://signal.salk.edu

2 http://arabidopsis.info

3 http://www.plantprobes.net/index.php

4 http://www.plantprobes.net/index.php

5 https://www.nikoninstruments.com/it_IT/

6 https://imagej.nih.gov/ij/

7 http://www.whatman.com/index.aspx

8 http://www.novozymes.com/

9 http://www.shodex.de/

10 http://www.malvern.com/
