# Supplementary material for: Dissecting Seed Mucilage Adherence Mediated by FEI2 and SOS5
Source: Front Plant Sci. 2016 Jul 29;7:1073. doi: 10.3389/fpls.2016.01073 (PMC4965450; doi:10.3389/fpls.2016.01073)
Supplement: Supplementary file 1 [file Table_1.PDF]

SUPPLEMENTAL TABLE 1: Primers used in this study.

Gene	Accession	Primer	Sequence
CESA5/MUM3	At5g09870	*cesa5-2* FP	GGAGTGGCCTTGAATCTGAA
		cesa5-2 RP	CGGGGTGAAGAATACGGTAA
MUM5/MUCI21	At3g10320	*mum5-2* FP	TTGGCCTCTTAGCCTTTCTTC
		mum5-2 RP	CGGGTTAAAACTTCTTGTGGG
SOS5/FLA4	At3g46550	*sos5-2* LP	GAAACTGGGAATAACCTTCGG
		sos5-2 RP	TTCTCGAGACCAAACCTC
FEI2	At2g35620	SAIL 150 A08 LP	TGCGTCGAGACTCTCTCTCTC
		SAIL 150 A08 RP	TGATGTTCCATGGAGATTTGC
SALK		LBb1.3	ATTTTGCCGATTTCGGAAC
WiscDsLox		P745	AACGTCCGCAATGTGTTATTAAGTTGTC
SAIL		SAIL LB 1	GCCTTTTGAGAAATGGATAAATAGCCTTGCTTC
pro*UBQ10*-*SOS5*-Citrine	At3g46550	UBQ10p-SacII-F	aaCCGCGGgtcgacgagtcagtaataaacg
		UBQ10p Sph1-R	aaGCATGCtgttaatcagaaaaactcagatta
		Sph1-FLA4-NSP-F	acatGCATGCAGATGGCGAACGTAATCTCAATT
		Xma1-FLA4-NSP-R	cccCCCGGGTGCGGCGGCGGTGGAAGA
		citrin-Xma-F	CCCCGGGTAATGGTGAGCAAGGGCGAG
		citrin-BsrGI-R	ACTTGTACAGCTCGTCCATG
		BsrGI-FLA4-F	ACATTGTACAAGATTAACGTCACCGCCGTCCTC
		Not1-FLA4-R	ataagaatGCGGCCGCTCATACCAAAACATAACAAAATG
		HSP18.2t/NotI-F	aaGCGGCCGCATATGAAGATGAAGATGAAATATTTG
		HSP18.2t/ApaI-R	aaGGGCCCATCTTATCTTTAATCATATTCCATAG
